# The One Health Action Plan Against Antimicrobial Resistance

**DOI:** 10.3390/antibiotics15101026

**Published:** 2026-10-15

**Authors:** Anna Maria Spagnolo, Maria Teresa Montagna, Osvalda De Giglio

**Affiliations:** 1 Department of Health Sciences, University of Genova, Via Pastore 1, 16132 Genova, Italy; 2 Interdisciplinary Department of Medicine, Hygiene Section, University of Bari Aldo Moro, 70124 Bari, Italy; mariateresa.montagna@uniba.it (M.T.M.); osvalda.degiglio@uniba.it (O.D.G.)

Antimicrobial resistance (AMR) represents a severe problem in public health due to the increasing spread amongst the general population, animals, and the environment of antibiotic-resistant microorganisms with resistance phenotypes extended to more than one class of antibiotics [1]. A worrying increase in microbial resistance has been observed in recent years, particularly among Gram-negative bacteria (especially Enterobacterales) [2,3,4,5,6].

The increasing emergence and spread of resistant microorganisms compromise the effectiveness of antimicrobial therapies, increase morbidity and mortality, and place a significant economic burden on healthcare systems worldwide.

In 2021, an estimated 4.71 million (95% UI 4.23–5.19) deaths were associated with bacterial AMR, with 1.14 million (1.00–1.28) deaths attributable [7]. The excessive and improper use of antimicrobials in humans, animals, and plants are the main factors responsible for developing drug-resistant pathogens. According to the WHO, without harmonized action on a global scale, the world is heading toward a post-antibiotic era in which common infections could kill again. It has been recognized that rising antimicrobial resistance seriously threatens the future management of current and emerging pathogens [8,9].

The complexity of AMR results from its multifactorial nature, which involves human medicine, veterinary practice, food production systems, environmental contamination, and global mobility. For this reason, addressing AMR requires a comprehensive and integrated “One Health” approach that recognizes the interconnectedness of human, animal, and environmental health. This recognition is the premise of the Global Action Plan on Antimicrobial Resistance, unanimously endorsed by WHO Member States in 2015 [10], which called for concerted action across all relevant sectors. The plan emphasizes collaboration among stakeholders in the development, implementation, and monitoring of programs, policies, regulations, and research activities, with the aim of mitigating antimicrobial resistance and achieving better health and economic outcomes.

This Special Issue of *Antibiotics* brings together a series of articles that explore various aspects of antimicrobial resistance, ranging from environmental reservoirs and regulatory frameworks to patterns of antimicrobial use and global factors that contribute to the spread of resistance.

Several contributions focus on the environmental dimension of antimicrobial resistance, which is increasingly recognized as a critical component in the AMR cycle [11].

Rogowska et al. [12] analyze pharmaceutical contamination of aquatic environments and the European regulatory framework for addressing it. The authors describe the main sources and pathways of pharmaceutical residues, including antibiotics, entering into aquatic ecosystems, such as livestock and agricultural activities, stormwater runoff, improper disposal of medications, and incomplete removal of drugs during wastewater treatment, which then facilitates their entry into surface waters. Their analysis highlights the evolution of the EU’s legislative framework, including updates to the Water Framework Directive and the “Watch List” system for monitoring emerging contaminants. These regulatory developments aim to improve the monitoring of pharmaceutical residues in aquatic environments and strengthen preventive strategies against AMR emergence in environmental reservoirs. The authors emphasize the importance of protecting aquatic environments from pharmaceutical contamination as part of efforts to combat antimicrobial resistance.

Food production is another setting in which antimicrobial use can promote the selection of resistant bacteria, which can reach humans through the consumption or handling of contaminated food. Spagnolo et al. [13] examine the spread of antimicrobial resistance along the food chain, drawing on data from livestock farming, aquaculture, and agriculture. The review describes how antimicrobial use in these sectors drives the selection of resistant microorganisms. Particular attention is given to animal production, where the extensive use of antimicrobials facilitates the selection of resistant bacteria. The practice of using inadequately treated manure as fertilizers can further spread these bacteria and their resistance genes to soil, water, and crops. The authors analyze the main foodborne resistant bacteria and the molecular mechanisms underlying resistance. They also examine regulatory measures and identify persistent gaps in surveillance, diagnostic capacity, and coordination among the human, veterinary, and environmental sectors.

To address these challenges, the review emphasizes the importance of a coordinated “One Health” approach that combines responsible antimicrobial stewardship with improvements in hygiene, biosecurity, and agricultural practices. Priorities include reducing the routine prophylactic use of antimicrobials, strengthening surveillance of resistance and antimicrobial consumption, and expanding access to rapid diagnostic tools to guide treatment. The authors also discuss the potential contribution of artificial intelligence and genomic technologies to surveillance, along with the need for new therapeutic options.

The need for new therapeutic options is also examined by Sora et al. [14], who emphasize that, to reduce and overcome antimicrobial resistance, at least three complementary and integrated actions must be taken: monitoring the resistome, developing protective strategies, and developing new treatments. The authors argue that these efforts must proceed hand-in-hand as part of a “One Health” approach, given the continuous adaptation of bacteria. Their discussion of the environmental resistome focuses on resistance genes in soil, water, and air, and on the use of molecular and genomic methods to monitor their distribution and spread. Prevention strategies include appropriate prescribing of antimicrobials, improved hygiene and biosafety practices, vaccination in livestock farms, and effective management of wastewater and pharmaceutical waste. The review also examines phage therapy, metal nanoparticles, and antimicrobial peptides as potential alternatives or complements to conventional antibiotics. Alongside their antibacterial properties, the authors show the limitations that may hinder their application, including the narrow spectrum of activity of phages, uncertainties regarding the long-term toxicity of nanoparticles, and the susceptibility of peptides to degradation in vivo. Artificial intelligence is also considered as a tool for identifying antimicrobial compounds and improving the activity of existing peptides, although its usefulness depends on the quality and availability of training data. By considering these approaches together, the review places therapeutic innovation within a broader strategy that also requires constant surveillance and prevention measures.

The global dynamics of the spread of antimicrobial resistance are further explored by Collignon and Beggs [15], who examine the role of international travel and tourism in the transmission of resistant bacteria. The authors have shown that in some countries, the incidence of AMR bacterial infections, among both visitors and returning travellers, is much higher than that observed in the resident population. Visitors from high-AMR regions can introduce resistant bacteria into host populations, while returning residents may import novel strains acquired abroad. Their analysis demonstrates that global mobility can act as a major vehicle for the spread of AMR microorganisms, facilitating their introduction across geographical borders. The authors underscore that environmental exposure during travel, particularly through contaminated water or food, may promote colonization even in individuals without direct exposure to antibiotics or contact with healthcare facilities. These findings reinforce the need for more effective surveillance systems and for integrating travel-related risk factors into strategies for preventing antimicrobial resistance.

Another important topic covered in this Special Issue concerns patterns of antimicrobial consumption and use, which remain among the main drivers of resistance development. Mudenda et al. [16] provide an in-depth analysis of antimicrobial consumption in Zambia, examining trends in both the human and animal health sectors between 2018 and 2023. Their findings reveal substantial antibiotic use in the human health sector, particularly penicillins, cephalosporins, and macrolides. According to the WHO’s AWaRe classification, 47% of antibiotics belonged to the “Access” group, 40% to the “Watch” group, and 3% to the “Reserve” group. In the animal health sector, tetracyclines were the most prevalent, followed by sulphonamides and penicillins. Overall, the study found high antimicrobial consumption and antimicrobial use, especially Watch-group antibiotics and tetracyclines, underscoring the importance of strengthening antimicrobial stewardship programs and regulatory oversight, and pointing to the need for integrated surveillance systems that encompass both human and veterinary medicine.

Taken together, the articles included in this Special Issue illustrate the multidimensional nature of antimicrobial resistance. Environmental contamination, antimicrobial consumption patterns, international travel, food production practices, and microbial evolution all interact to shape the global landscape of antimicrobial resistance. These studies highlight that no single intervention will be sufficient to address the problem. Instead, coordinated strategies involving policymakers, healthcare professionals, researchers, veterinarians, environmental scientists, and public health authorities are essential.

In particular, the “One Health” framework emerges as a unifying theme across all the articles presented in this Special Issue. By integrating the perspectives of human health, animal health, and environmental sciences, the “One Health” approach provides a comprehensive strategy for addressing AMR at its sources and along its transmission pathways. Strengthening surveillance systems, promoting the responsible use of antimicrobials, improving environmental monitoring, and investing in innovative therapeutic approaches will be crucial components of future efforts to control antimicrobial resistance.

In conclusion, antimicrobial resistance remains a rapidly evolving global challenge that requires urgent and coordinated action. Ongoing interdisciplinary collaboration, supported by robust regulatory frameworks and sustained investment in research and surveillance, will be essential to safeguarding the effectiveness of antimicrobial therapies and protecting global public health.

## Data Availability

No new data were created or analyzed in this study. Data sharing is not applicable to this article.
